# Heterogeneity of tremor mechanisms assessed by tremor-related cortical potential in mice

**DOI:** 10.1186/s13041-015-0093-2

**Published:** 2015-01-15

**Authors:** Young-Gyun Park, Jee Hyun Choi, Chungki Lee, Sehyun Kim, Youngsoo Kim, Ki-Young Chang, Sun Ha Paek, Daesoo Kim

**Affiliations:** Department of Biological Sciences, Korea Advanced Institute of Science and Technology (KAIST), 335 Gwahak-ro, Yuseong-gu, Daejeon, 305-701 Republic of Korea; Center for Neural Science, Division of Life Science, Korea Institute of Science and Technology, Seoul, 136-79 Republic of Korea; Department of Physics, KAIST, Daejeon, 305-701 Republic of Korea; Department of Medical Science and Engineering, KAIST, Daejeon, 305-701 Republic of Korea; Department of Neurosurgery, Hypoxia/Ischemia Disease Institute, Seoul National University College of Medicine, Seoul, 110-744 Republic of Korea

**Keywords:** Tremor mechanism, Cortical rhythm, Harmaline, T-type Ca^2+^ channels

## Abstract

**Background:**

Identifying a neural circuit mechanism that is differentially involved in tremor would aid in the diagnosis and cure of such cases. Here, we demonstrate that tremor-related cortical potential (TRCP) is differentially expressed in two different mouse models of tremor.

**Results:**

Hybrid tremor analysis of harmaline-induced and genetic tremor in mice revealed that two authentic tremor frequencies for each type of tremor were conserved and showed an opposite dependence on CaV3.1 T-type Ca^2+^ channels. Electroencephalogram recordings revealed that α1^−/−^;α1G^-/-^ mice double-null for the GABA receptor α1 subunit (Gabra1) and CaV3.1 T-type Ca^2+^ channels (Cacna1g), in which the tremor caused by the absence of Gabra1 is potentiated by the absence of Cacna1g, showed a coherent TRCP that exhibited an onset that preceded the initiation of behavioral tremor by 3 ms. However, harmaline-induced tremor, which is known to be abolished by α1G^−/−^, showed no TRCP.

**Conclusions:**

Our results demonstrate that the α1^−/−^;α1G^−/−^ double-knockout tremor model is useful for studying cortical mechanisms of tremor.

## Background

Tremor is an involuntary rhythmic oscillation of body parts [1,2]. Various mechanism ranges from reflex loop to central oscillation has been proposed for the origin of oscillatory activity generating tremor [1,2]. Animal models of tremor have been contributed to examine those hypotheses.

Harmaline is a plant-derived metabolite used to induce tremor in animals [3-5]. Harmaline tremor is an action-induced tremor and coherent with pathological oscillation in olivocerebellar pathway, which propagates to reticulospinal pathway and entrains muscles with synchronized oscillation [4,6,7]. Either knockout of the *Cacna1g* gene encoding the Ca_V_3.1 channel α1G subunit (α1G^−/−^ mice) or pharmacological blockage of T-type Ca^2+^ channels in the inferior olive abolishes 10–15 Hz harmaline-induced tremor and the pathological oscillation [8], suggesting that activity of T-type calcium channel is critical for generating harmaline tremor.

T-type calcium channel has a contradictory role in the other model of action-induced tremor. In Gabra1 model of action tremor, knockout mice of α1 subunit of the γ-aminobutyric acid (GABA) receptor, additional knockout of α1G enhances action-induced tremor [9]. Considering that T-type calcium channel is involved in thalamocortical oscillations [10,11] and cortical lesion has no effect on harmaline tremor [7,12], we hypothesized that two tremor models has different involvement of cortical mechanisms.

Here, using pharmacogenetic dissection of tremor frequencizes and high-resolution electroencephalograzphic (EEG) techniques [13] combining with event-related potential analysis(ERP) [14-17], we reveal that abnormal cortical rhythmicity in the form of tremor-related cortical potential (TRCP) is differentially expressed between harmaline and genetic models of action tremor.

## Results

### Pharmacogenetic determination of tremor frequency independence

To test the independence of mechanism between harmaline and genetic tremors (α1^−/−^ and α1^−/−^;α1G^−/−^) [9], we performed pharmacogenetic experiment in which two types of tremor were produced in a mouse. We predicted two possibilities. The first is that the two tremor mechanisms interact to generate a hybrid tremor according to the dominant/recessive relationship between them. Alternatively, the two tremor frequencies may emerge independently, like the difference in behavior between two genes on separate chromosomes.

**Figure 1:**
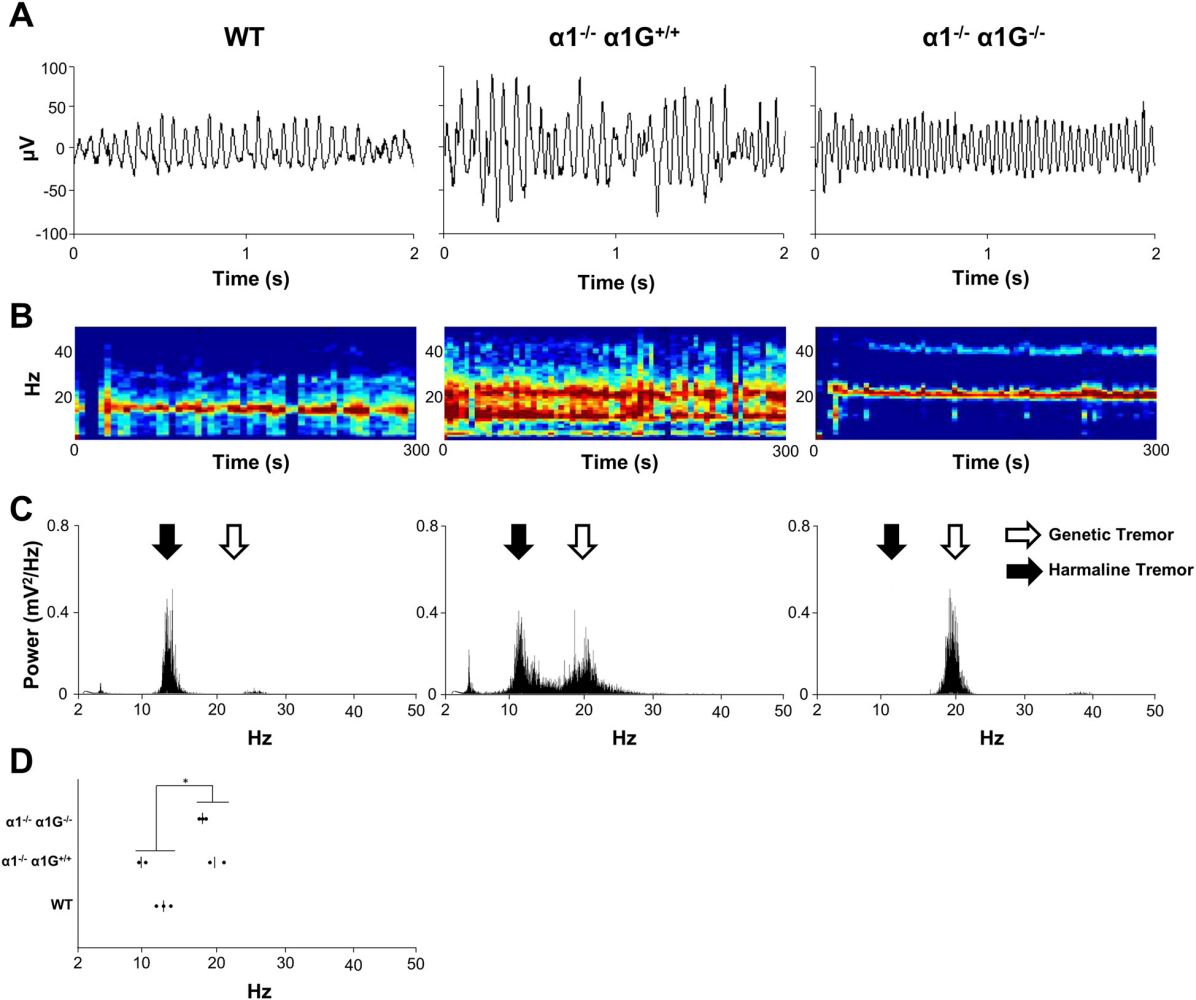
**Functionally distinct tremorogenesis in harmaline tremor and α1** ^**-/-**^ **;α1G** ^**−/−**^ **tremor. (A-C)** Tremor after harmaline (15 mg/kg) injection into WT (*left*), α1^−/−^ (*center*), and α1^−/−^;α1G^−/−^ (*right*) mice (n = 3 per genotype) are shown as accelerometer signal **(A)**, spectrogram **(B)**, and power spectral analysis **(C). (D)** Comparison of tremor frequencies in WT (*left*), α1^−/−^ (*center*), and α1^−/−^;α1G^−/−^ mice. T-test, *P < 0.05.

After harmaline administration (9 mg/kg), wild-type (WT) mice exhibited only 10–15 Hz tremor (Figure 1A-C, *left*), whereas α1^−/−^;α1G^−/−^ mice showed only 15–30 Hz tremor (Figure 1A-C, *right*). In contrast, harmaline-treated α1^−/−^ mice exhibited two separable band frequencies, 10–15 and 15–30 Hz, reflecting the coexistence of authentic harmaline and genetic tremor (Figure 1A-C, *middle,* and Figure 1D). Considering that the different tremor frequencies reflect the involvement of different neural circuit mechanisms [1,2], the two action tremor models appear to have distinct neural circuit mechanisms.

### Tremor-related cortical potentials in α1^−/−^;α1G^−/−^ mice

**Figure 2:**
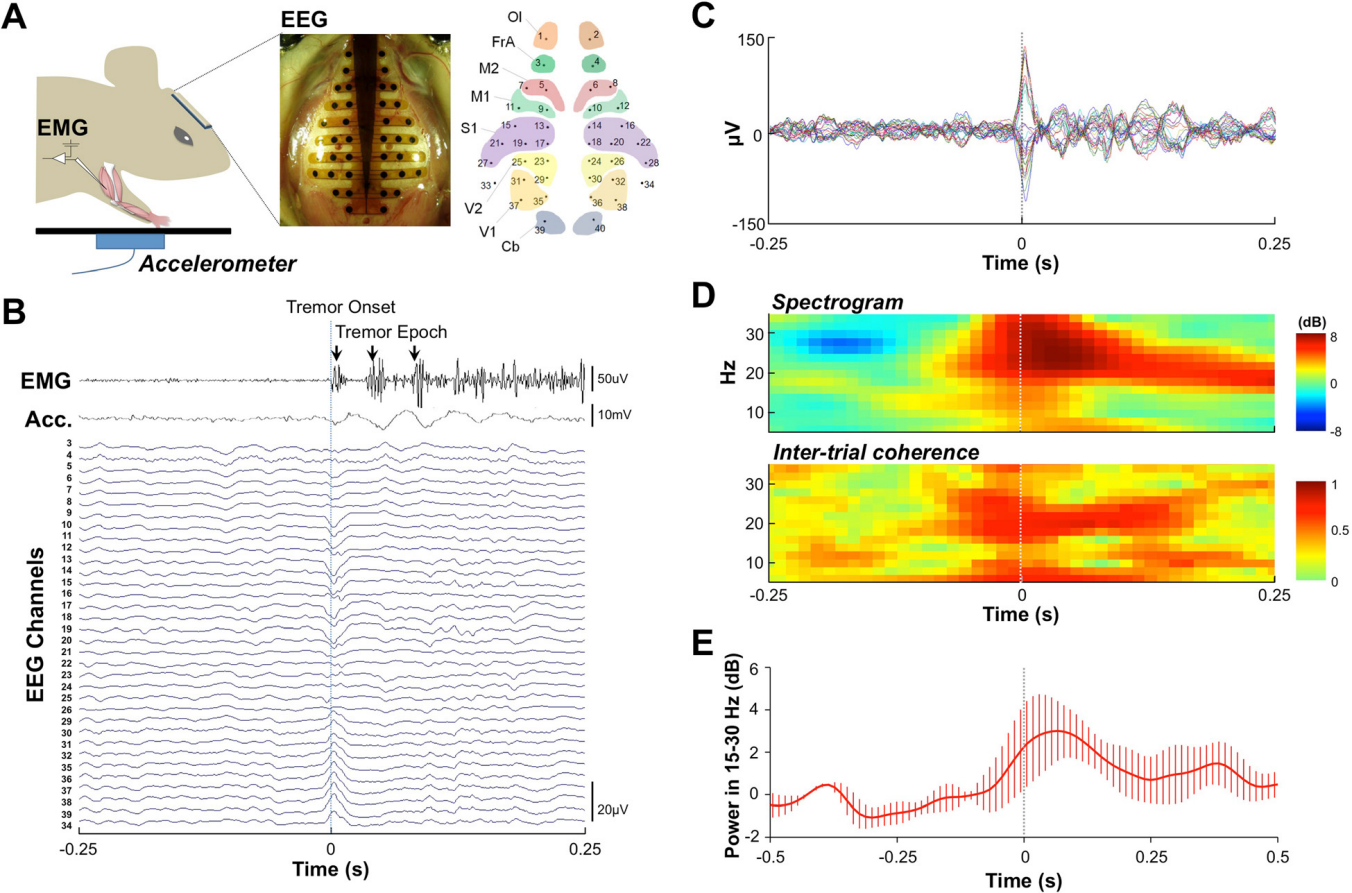
**Tremor onset-related cortical potential in α1** ^**−/−**^ **;α1G** ^**−/−**^ **mice. (A)** Schematic diagram of EEG, EMG, and accelerometer recordings during mouse behavior. EMG activity was recorded from triceps brachii of left and right forelimbs. Topography of the high-resolution EEG array attached to the skull (*middle*) and locations of channels (*right*). The topography of the high-resolution EEG array was adapted with permission from Lee et al. (2011) [18]. **(B)** Representative EEG, EMG, and accelerometer (Acc.) traces upon α1^-/-^;α1G^−/−^ tremor onset. The EMG shown is from the right forelimb. **(C)** ERPs of tremor onset. Different colored traces indicate averaged activity of different channels. **(D)** Spectrogram (*top*), inter-trial coherence (*bottom*), and **(E)** 15–30 Hz power of ERPs from the left S1 (n = 3) during tremor onset. Ol, olfactory cortex; FrA, frontal association cortex; M2, secondary motor cortex; M1, primary motor cortex; S1, primary somatosensory cortex; V2, secondary visual cortex; V1, primary visual cortex; Cb, cerebellar cortex.

Using high-resolution EEG covering the whole frontal and occipital cortex (Figure 2A) and forelimb electromyography (EMG), we measured the cortical activity associated with forelimb tremor in α1^−/−^;α1G^−/−^ mice. We isolated cortical potentials associated with tremor onset by averaging EEG activity corresponding to the first discharge of each tremor epoch. Tremor onset-related potentials in α1^−/−^;α1G^−/−^ mice initiated approximately 3 ms prior to behavioral tremor onset and were followed by 15–30 Hz oscillations corresponding to the accompanying muscular tremor discharges (Figure 2B and C). The resulting TRCPs were phase-locked to the 15–30 Hz tremor (Figure 2B and C), although their amplitudes and phases were variable among channels (Figure 2B and C), indicating the differential involvement of cortical subdomains. Power spectral analyses also confirmed that TRCPs were initiated upon tremor onset (Figure 2D, *upper*, and 2E) and coincided with tremor events (Figure 2D, *lower*). These results indicate that the α1^-/-^;α1G^−/−^ tremor includes the cortical circuit mechanism, although the origin remains unknown.

### No TRCPs in harmaline tremor

**Figure 3:**
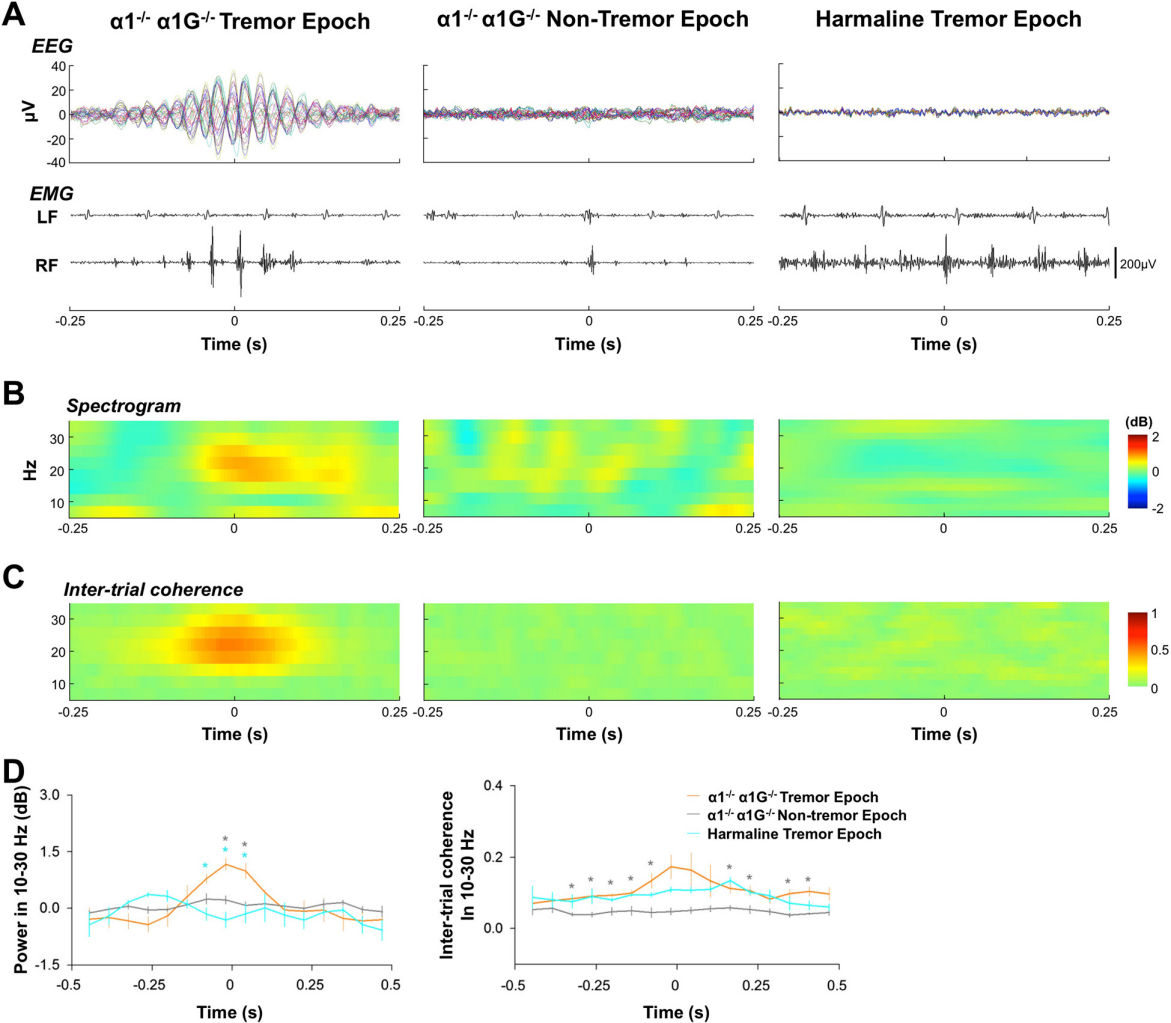
**Comparison of tremor-related activity between two essential tremor models. (A)** ERPs (*top*) and right forelimb (RF) EMG activity (*bottom*) upon α1^−/−^;α1G^−/−^ tremor discharge (*left*), α1^−/−^;α1G^−/−^ non-tremor discharge (*center*), and harmaline tremor discharge (*right*). Arrows indicate each tremor discharge. Spectrogram **(B)** and inter-trial coherence **(C)** for the EEG channel on the left S1. For A to C, 100 events were randomly chosen from among all events for comparisons between ERPs. **(D)** 10–30 Hz power (*left*) and inter-trial coherence (*right*) for the EEG channel on the left S1 (n = 3 for each). Asterisks indicate comparisons between α1^−/−^;α1G^−/−^ tremor and harmaline tremor (light blue), and between tremor and non-tremor in α1^-/-^;α1G^−/−^ mice (gray). *P < 0.05 (two-tailed t-test).

We then compared TRCPs in the two tremor models. When we aligned and averaged EEG traces along the timing of tremor discharges in α1^-/-^;α1G^−/−^ mice, TRCPs were obtained as an oscillatory pattern (Figure 3A, *left*). However, TRCPs were not found in harmaline tremor (Figure 3A, *right*) or in non-tremor contractions of α1^−/−^;α1G^−/−^ mice (Figure 3A, *middle*). The power and coherence of TRCPs were significantly lower in harmaline tremor than in α1^−/−^;α1G^−/−^ tremor (Figure 3B-D). These results suggest that TRCPs can be a robust measure for discriminating these two types of tremor models.

## Discussion

Essential tremor is a neurological condition characterized by action-induced tremors [2,19]. Considerable evidence suggests that essential tremor is heterogeneous with respect to affected body parts, tremor frequency, and drug sensitivity [20-24]. The heterogeneity appears to reflect the involvement of different neural substrates. While some studies have reported that abnormal cortical rhythms are associated with tremor in a subset of essential tremor patients [25,26], another clinical study failed to find such a correlation [27], necessitating in-depth comparative studies on cortical involvement in the generation of essential tremor [25-28].

Two action-induced tremor models used in present study has been proposed as an animal models of essential tremor [4,9]. By employing event-related potential analysis, we identified TRCPs in α1^-/-^;α1G^-/-^ mice. However, we failed to find out tremor-related activity in harmaline tremor, suggesting that two models of tremor have different cortical dependency.

Whereas harmaline and α1^−/−^ tremors are ameliorated by propranolol [29,30], α1^−/−^;α1G^−/−^ tremor is resistant to propranolol [9]. Deuschl et al. reported that the patients with propranolol susceptibility show synchronous activity between agonist and antagonist muscles during tremor, whereas the patients with propranolol resistance exhibit reciprocal antagonist muscle activity [20]. Notably, harmaline tremor, which is sensitive to propranolol [30], also shows co-contraction of agonist and antagonist muscle pairs during tremor [31]. Thus, it is plausible that the TRCP serves a role in generating propranolol-resistant or reciprocal agonist–antagonist contracting subtypes of essential tremor. A comparative study on the cortical activity of essential tremor patients with different phenotypes would effectively test this hypothesis.

The two essential tremor models in this study are also different with respect to their underlying neural circuit mechanism. The Ca_V_3.1 channel is expressed in various motor pathways and may serve different functions [32]. In the thalamocortical pathway, where both *Gabra1* and *Ca*_*V*_*3.1* genes are co-expressed [33], Ca_V_3.1 may play an inhibitory role in tremorogenesis. In contrast, Ca_V_3.1 is involved in the generation of tremor in the inferior olive nucleus [8,32]. Inferior olive neurons are connected to muscles through the cerebelloreticulospinal pathway [6,34]. This means that harmaline could induce tremor while bypassing the involvement of the thalamocortical pathway. Indeed, evidence shows that neither the thalamus nor the motor cortex is associated with harmaline-induced tremor [7,12].

The TRCP in α1^-/-^;α1G^-/-^ mice is not merely a sensory feedback or a movement artifact for a number of reasons. First, it occurred prior to the onset of tremor, at least in some channels, suggesting the possibility that TRCPs are involved in the mechanism responsible for tremor onset (Figure 2). Moreover, TRCPs were not observed in harmaline tremor, which have an intensity similar to that of α1^−/−^;α1G^−/−^ tremor (Figure 3). Although tremoring muscle is known to provide sensory feedback to the cortex, it is not likely that our method is sensitive enough to detect the low level of associated neural activity. These TRCP findings suggest that the two types of tremor are differentially associated with physiological and neural circuit mechanisms.

In conclusion, we have demonstrated the presence of TRCP only in a certain animal model of essential tremor, suggesting its mechanistic heterogeneity. The TRCP characterized in our study could be a useful readout for the diagnosis of essential tremor type. Notably, in patients showing a high level of TRCP, therapeutic strategies that target thalamocortical pathways, rather than T-type Ca^2+^ channel blockers, might be effective. Furthermore, the α1^−/−^;α1G^−/−^ mouse should be a useful model for revealing how the cerebral cortex can generate tremor.

## Methods

### Animals

Animal care and handling conformed to the institutional guidelines of the Animal Care and Use Committee of the Korea Advanced Institute of Science and Technology (KAIST). Mice heterozygous for GABA_A_ receptor α1 deletion (α1^+/−^) in a C57BL/6 J genetic background were obtained from Dr. Gregg E. Homanics at the University of Pittsburgh. Crossing α1^+/−^ and α1G^+/−^ mice yielded double-heterozygous animals (α1^+/−^;α1G^+/−^), which were mated to produce WT (α1^+/+^;α1G^+/+^), α1 mutant (α1^−/−^;α1G^+/+^), and double-mutant (α1^−/−^;α1G^−/−^) mice. WT mice were utilized for the harmaline-induced tremor model. All experiments were performed using adult mice (>10 weeks old). Mice were housed under a 12-hour light/dark cycle (7 a.m. to 7 p.m.) with free access to water and food.

### High-resolution EEG

EEG electrode arrays were prepared as previously described [13]. For surgical anesthesia, ketamine (100 mg/kg) mixed with xylazine (5 mg/kg) was administered intraperitoneally. After surgical anesthesia had developed, the head of the mouse was fixed on a stereotaxic apparatus (David Kopf Instruments, CA, USA), and an incision of ~2.5 cm was made on the scalp. The skull was exposed using micro-clamps, and tissue debris was removed by carefully wiping with a 70% ethanol-soaked cotton ball. After moisturizing the skull surface with a drop of tap water, a polyamide-based, high-density film electrode was placed on the skull and carefully positioned so that the vertical midline was aligned with the skull midline and the bregma met the upper edge of the third branch. After positioning the electrode, two screws were implanted onto the skull, and dental acrylate was coated over the electrode array. The viscosity of the dental acrylate mix used was carefully examined before application. Mice were allowed to recover under an infrared lamp and housed singly until the experiment.

### Electromyography

The electrodes for electromyography (EMG) in mice were made with Teflon-coated stainless steel wires (A-M Systems, WA, USA) and implanted as previously described [35]. Briefly, two wires were combined and twisted together with a knot 4 cm from the end. The Teflon insulation was removed from 0.5-mm-long segments 1 or 2 mm from the knot in both wires to create two exposures located 1 mm apart. The ends of both wires were crumpled into the shaft of a 26-gauge needle. The opposite ends of the wires were cut ~8 cm from the knot, bared, and soldered to a connector. After the fur in the dorsal neck region was shaved away, small incisions were made in the skin above the left and right forelimb muscles (triceps brachii). EMG electrodes were inserted under the skin from the neck incision to the muscles. The needle harboring the electrode was inserted into the target muscle until the knot proximal to the bared regions was firmly butted against the surface of the muscle. The distal end of the electrode exiting the muscle was loosely knotted, and the knot was moved to the muscle surface and tightened. Incisions in the limbs were closed with silk suturing material.

### Analysis of tremor

After habituation in the recording room for 30 minutes, the mouse was placed on a stainless steel plate (25 cm diameter) suspended by two elastic rubber bands. The edge of the plate was surrounded by paper (5 cm wide) to prevent mice from falling off. The behavior of the mouse was monitored using a video camera positioned above the platform. For induction of harmaline tremor, harmaline hydrochloride dissolved in 0.9% saline was injected intraperitoneally (15 mg/kg). A DC response accelerometer (Model No. 3711D1FA3G; Piezotronics, NY, USA) was mounted below the steel plate to aid in tremor detection.

### Acquisition of EEG and EMG signals

High-resolution EEG, EMG, and accelerometer signals were obtained using a SynAmps^2^ amplifier (Neuroscan Inc., TX, USA) with a 2000-Hz sampling rate. Channel 34 of the EEG was used as a reference and channel 33 was used as a ground. Contact impedances of EEG channels were measured using Scan 4.3 software (Neuroscan Inc.) with 30-Hz test pulses. EEG channels with abnormal impedance (>500 kOhm) were excluded from further analyses.

### Analysis of tremor-related potentials

#### Pre-processing

All signal processing and analyses were performed using the Matlab-based open source toolbox EEGLAB [36] or custom Matlab codes. Signals were loaded onto EEGLAB and filtered with a 100–Hz high-pass filter for EMG signals and a 1–200 Hz band-pass filter for EEG and accelerometer signals. All filters used were finite-impulse response filters. EEG signals were re-referenced by common average reference using an EEGLAB plug-in algorithm.

#### Tremor detection

EMG traces obtained from the left or right forelimb triceps brachii muscle were used for measuring tremor. Raw EMG traces were rectified and smoothed using a moving-average window. A cluster of EMG action potentials with peak amplitudes 2.5-fold greater than the standard deviation of baseline EMG activity and separated from others by more than 25 ms was defined as an ‘EMG discharge’. Rhythmic and successive EMG discharges in a specific frequency range (15–30 Hz for α1^−/−^;α1G^−/−^ and 10–15 Hz for harmaline tremor) were identified as a ‘tremor epoch’. Each EMG discharge in a tremor epoch was defined as a ‘tremor discharge’. The ‘tremor onset’ was determined as the onset time of the first tremor discharge in a tremor epoch. Irregular EMG discharges temporally separated from the tremor epoch by more than 1 second in α1^−/−^;α1G^−/−^ mice were identified as ‘non-tremor discharges’. Tremor onset is used for ERP analysis in Figure 2. However, we used tremor discharge itself for comparing TRCP in two tremor models (Figure 3) because single tremor epoch is much longer in harmaline tremor so that it was hard to collect enough number of tremor epochs for ERP analysis. Event detection was performed in the left and right forelimb independently using Matlab codes, and was confirmed by visual inspection.

#### Event-related potential analysis

EEG channel activity during each event (500 ms pre- and post-event) was collected, and a baseline correction was applied to the pre-event period for each event and channel. For the calculation of ERPs, the waveforms of collected events (>100 for tremor discharge and non-tremor discharge, and >50 for tremor onset) were averaged, and the ERP from each channel was plotted in a different color. ERPs of tremor onset, tremor discharge and non-tremor discharge for α1^−/−^;α1G^−/−^ and harmaline tremor models were calculated, and ERPs of tremor onset and tremor discharge were identified as TRCPs. Tremor events that occurred in only one forelimb at a given moment (>0.5 seconds apart from contralateral limb tremor events) were used for ERP calculations. Inter-trial coherence is a frequency-domain measure of activity synchronization between event-related trials [36]. Inter-trial coherence value indicates degree of consistency of oscillatory phase across trials at certain latency and frequencym [37] and calculated by EEGLab [36].

### Statistical analysis

All data are presented as mean values ± SEM, and differences between experimental groups were analyzed for statistical significance using Sigmastat 3.1.

## Abbreviations

α1^−/−^: mice null for Gabra1
α1^-/-^;α1G^-/-^: mice double-null for Gabra1 and Cacna1g
EEG: Electroencephalography
EMG: Electromyography
ERP: Event-related potential
TRCP: Tremor-related cortical potential
